# Is Physical Activity Protective against Emotional Eating Associated Factors during the COVID-19 Pandemic? A Cross-Sectional Study among Physically Active and Inactive Adults

**DOI:** 10.3390/nu13113861

**Published:** 2021-10-28

**Authors:** Marcela Larissa Costa, Maycon George Oliveira Costa, Márcia Ferreira Cândido de Souza, Danielle Góes da Silva, Diva Aliete dos Santos Vieira, Raquel Simões Mendes-Netto

**Affiliations:** 1 Postgraduate Program in Nutrition Sciences, Federal University of Sergipe, São Cristóvão 49100-000, Brazil; marcelaa.costa.nutricionista@gmail.com (M.L.C.); danygoes@yahoo.com (D.G.d.S.); 2 Laboratory of Studies in Nutrition and Exercise, Federal University of Sergipe, São Cristóvão 49100-000, Brazil; maycongeorge10@gmail.com; 3 Nutrition Clinic, University Hospital of Federal University of Sergipe, Aracaju 49060-676, Brazil; nutrimarciacandido@gmail.com; 4 Nutrition Department, Federal University of Sergipe, São Cristóvão 49100-000, Brazil; diva.nutricao@gmail.com

**Keywords:** physical activity, eating behavior, COVID-19, eating habits, body image, life stress

## Abstract

Physical activity levels during the COVID-19 pandemic have been decreasing and this may be a risk factor for development of emotional eating and its associated factors. The aim of the study was to analyze the factors associated with emotional eating among individuals with different physical activity levels during the COVID-19 pandemic. Data relating to the pandemic on physical activity, emotional eating, sociodemographic data, perceptions about lifestyle habits, body satisfaction, and perceptions about eating habits and food consumption were collected. Factors associated with emotional eating in the group of active and inactive individuals were observed using multiple linear regression controlled for age, sex, BMI, and monthly income. Emotional eating for the active group was associated with perceived stress, body dissatisfaction, and increased consumption of sweets and desserts. In addition to these factors found among the active group, working or studying >8 h/day, sleep worsening, increased amount of food consumed, increased purchase of food through delivery, and increased vegetable consumption were also associated with emotional eating for the inactive group. These findings suggest a potential protective role of physical activity in the appearance of factors associated with emotional eating during the COVID-19 pandemic.

## 1. Introduction

The impact of physical activity on health is irrefutable, as it is associated with an improvement in health and well-being, a reduction in the risk of more than 25 non-communicable chronic diseases (NCDs), and a reduction in premature mortality [1,2,3,4,5]. In addition, previous studies have shown that regular moderate physical activity can increase immune health, promoting protection against infections caused by intracellular microorganisms [6,7].

There was a change in routine and social distancing became part of the new daily life of the population due to the pandemic caused by the coronavirus (COVID-19) [8]. If on the one hand this is the best option and recommendation to stop the rapid advance of the virus, on the other hand it brings with it a change in lifestyle with direct consequences to the health behavior of individuals. Such behaviors generally include certain levels of physical activity [9]. Studies show a considerable reduction in physical activity levels in Brazil and worldwide during the pandemic [10,11,12], with the concern about exposure to the virus being one of the causes [13].

Along with physical inactivity, narrowing the boundaries between work and personal life is a significant challenge during the pandemic [14,15]. These factors together have the potential to act in synergy to worsen mental health, constituting another dimension of health which has been negatively affected in this period [16]. Studies point to alarming pandemic-related stress data [17,18,19], with high prevalence in Brazil and worldwide [20,21]. This in turn has a negative influence on practicing physical activity [22,23]. Thus, the high prevalence of physical inactivity becomes a risk factor for developing stress, and stress becomes a risk factor for physical inactivity [24].

Another factor closely associated with both stress, sedentary lifestyle, and physical activity is body dissatisfaction [25,26,27]. Body dissatisfaction rates increased during the pandemic [28,29], with this factor directly influencing the eating behavior of individuals [30], and being associated with food consumption based on ultra-processed foods, soft drinks, sweets, and candy [27,31].

Emotional eating (EE) is a behavior defined by eating as a result of emotions, with or without the stimulus of hunger. When the rational function is inhibited, the emotional function predominates, leading to food disinhibition [32,33,34]. Emotional eaters choose these foods as a coping or healing strategy to manage their emotional state [35,36]. This disinhibitory state was previously associated with less physical activity [37,38]. However, it is known that physical activity practice is recognized as a protective factor for triggering this behavior [39,40,41].

EE during the pandemic was associated with negative eating habits, increased number of meals, consumption of sweets, fast food, and fat [42,43]. Understanding eating behavior and its possible associated factors is important to broaden the understanding of what can determine the population’s food consumption, since consumption rich in energy-dense and ultra-processed foods harms the population’s health and leads to a greater risk for developing NCDs [44], which are recognized risk factors for worsening COVID-19 infection [45,46,47].

No study to date has demonstrated the factors associated with EE between physically active and inactive individuals separately during the pandemic, considering that physical activity levels have been declining and this could be a trigger factor for developing EE. We hypothesized that active individuals would demonstrate fewer factors associated with EE than inactive individuals. Thus, the aim of the present study was to analyze the factors associated with EE among physically active and inactive individuals during the COVID-19 pandemic.

## 2. Materials and Methods

### 2.1. Study Design and Data Collection

A cross-sectional web-based online survey was carried out between December 2020 and January 2021. The survey link was distributed via academic/department e-mails of a public university in northeast Brazil and social media (Whatsapp and Instagram) using a snowball technique.

The questionnaire consisted of 73 objective questions in Portuguese divided into 6 sections. It required approximately 20 min to complete. Only 43 questions were used for the purpose of this manuscript objective. Data from completed forms were imported into a Microsoft Excel spreadsheet. The survey obtained 643 responses, of which 40 were excluded due to duplicate responses and 5 were excluded due to invalid answers. The study was performed in compliance with the Helsinki Declaration Guidelines and the project received ethical approval from an Ethic Committee under number: 4380553.

### 2.2. Participants

The study participants needed to be adults (18 to 59 years) and residents in the Brazilian territory to be included. Pregnant women were not eligible to participate. The sample size was estimated with the G power software program [48] for multiple linear regression with 19 potential predictor variables using medium effect size, *p*-value < 0.05 and power = 0.95, which indicated that a minimum of 217 participants were required in each regression.

### 2.3. Measures

#### 2.3.1. Physical Activity Practice during the Pandemic

Participants were initially asked about practicing physical activity, and two more questions were additionally asked about the frequency and total time of practice for those who were currently practicing physical activity during the pandemic, based on the “ConVid—Behavior Survey”, a national web survey by the Oswaldo Cruz Foundation [49]. The physical activity level during the pandemic was classified using the recommendation of 150 min/week [50], which was calculated using the mean point of frequency and duration in each category, as performed elsewhere [11,51]. For the analysis, participants were divided into two groups: “Physically active during the pandemic” (≥150 min/week) and “Physically inactive during the pandemic” (<150 min/week).

#### 2.3.2. Emotional Eating

The emotional eating behavior was assessed by the three-factor eating questionnaire subscale (TFEQ-R21) validated for Brazilian Portuguese [34]. The subscale was from a shortened questionnaire version [52] of the original 51-question TFEQ [53].

The emotional eating scale has 6 items and measures the propensity to overeat in response to negative emotional states, such as stress, anxiety, and depression. The scoring analysis of the questionnaire was performed according to the guidelines of the authors. Item scores ranged from 1 to 4 and the EE subscale items were subsequently added to calculate the mean; this result is called the raw score. The raw score was then converted using formulas for standardization of results and comparison with other studies using this methodology. The final score was calculated using the following formula: Behavior score: 100 * ((Raw mean of the subscale—Minimum raw score)/Maximum raw score). The values of the final score range from 0–100 and a higher score indicates greater EE.

#### 2.3.3. Sociodemographic Data

Participants were required to respond about their age, sex, education level, marital status (married, single or other), and state and city of Brazilian territory to assess sociodemographic characteristics. State and city information were used to describe the sample: the states collected were categorized into Brazilian regions (northeast, southeast, or other); the cities were used to describe if the individual lived in the capital or other cities. For the purpose of analysis, the educational level was categorized into Higher education (post-graduate and graduate degree) and Lower education (high school and below).

Monthly household income was assessed according to the criteria of socioeconomic strata of ABEP (2019): A: Up to USD $4886.48 or more; B1: Up to USD $2156.79; B2: Up to USD $1078.79; C1: Up to USD $589.81; C2: Up to USD $334.36; D: Up to USD $137.64 [54]. The strata were divided into two categories for analysis: Higher income (strata A to B2) and Lower income (strata C1 to D). Hours of work or study per day were also assessed: <4 h, 4–6 h, 6–8 h, >8 h/day were also assessed. The number of hours worked or studied was categorized into: Up to 8 h/day and More than 8 h/day.

Participants were also asked to self-report weight and height. These measurements were used to calculate the Body Mass Index (BMI) using the following formula: BMI = Weight (kg)/(height)^2^ (m). BMI was used in the analyses as a continuous variable.

#### 2.3.4. Perceptions on Life Habits during the Pandemic

Next, the participants were asked, “How do you rate your level of social isolation during the pandemic?” to assess the level of social isolation, with the possible answers being: (a) I do not leave home; (b) I leave 1–2 times/week; (c) I leave 3–4 times/week; (d) I leave 5–6 times/week; and (e) It is not possible to do social isolation in my routine. The responses were categorized into: High level of social isolation (not leaving home and leaving 1–2 time/week) and Low level of social isolation.

In addition, two other questions were asked to assess the perception of stress level and changes in sleep during the pandemic. The questions were: “Have you noticed a difference in your stress level during the pandemic?” and “Have you noticed any difference in your sleep during the pandemic?” All questions were answered using a 5-point Likert scale. The answers were dichotomized for analysis purposes.

#### 2.3.5. Perception on Body Satisfaction

In this section, two questions were asked about perception on body satisfaction and attempt to lose weight in the last six months. The first questions was, “Do you currently feel satisfied with your body?” The question was answered using a 5-point Likert scale. Moreover, a final question was asked: “Have you attempted to lose weight in the last 6 months?” with the option of answering “yes” or “no”.

#### 2.3.6. Perception on Eating Habits and Food Consumption during the Pandemic

Participant perception on eating habits during the pandemic was evaluated by four questions. The questions were: “In your opinion, your eating habits during the pandemic have been”; “Have you noticed a difference in the amount of food you are consuming during the pandemic?”; “Have you noticed a difference in the frequency of food preparation at home during the pandemic?” and “Have you noticed a difference in the frequency of purchasing ready-to-eat food via delivery during the pandemic?”. All questions were answered using a 5-point Likert scale. The answers were also dichotomized for analysis purposes.

Finally, the participants answered questions about perception of changes in food consumption during the pandemic. The list of foods was adapted from the food frequency questionnaire (FFQ) used in the ISACAMP-Nutri [55]. The food categories evaluated were (1) Vegetables; (2) Fresh fruit; (3) Refined cereals; (4) Sweets and desserts; and (5) Fast Food. For the five food categories, participants were asked whether consumption: (a) increased, (b) remained unchanged, or (c) decreased.

### 2.4. Pre-Test

The questionnaire developed by the authors underwent a pre-test before starting the data collection. The initial questionnaire was analyzed by the research group to help strengthen the available questions and answers. After the research group assessment, items from the sociodemographic questionnaire and the perception on food consumption during the pandemic questionnaire were modified to improve the general understanding. Next, a pilot questionnaire was applied to the target population in order to verify whether the elaborated questionnaire answered the following questions: clarity and precision of the terms, quality of the data and feedback. Thus, the questionnaire underwent subsequent corrections of the flaws and difficulties encountered by the participants.

The invitation to respond to the pilot questionnaire was carried out via dissemination by the Google Forms link on social networks and in contact with national researchers in the area. The results were extracted to a Microsoft Excel table and the data were interpreted and analyzed according to the research objective. A total of 340 responses were obtained, and after analyzing and discussing the questions and answers collected there was a need to reformulate the “perception on life habits during the pandemic”, “perception on body satisfaction”, and “eating habits during the pandemic” sections to improve the order of responses, improve the flow of questions, and standardize technical terms present in the questions. After the responses were reorganized, the questionnaire underwent a new analysis by the research group for final application.

### 2.5. Statistical Analyses

Data were analyzed using the Statistical Package for Social Sciences (SPSS) version 25.0. Continuous variables were subjected to the Kolmogorov-Smirnov test to verify the assumptions of normality. A descriptive analysis was performed using absolute numbers and percentages for categorical variables and mean and standard deviation or 95% confidence intervals for continuous variables. The Mann-Whitney test was used for comparison of continuous variables between physically active and physically inactive during the pandemic groups and the Chi-squared test was used to assess the association of categorical variables.

Two multivariate analyses by linear regression were performed using EE of physically active group and EE of physically inactive group as dependent variables. The independent variables used were all dichotomic from the sociodemographic data, perceptions on life habits during the pandemic, perceptions of body satisfaction, perceptions about eating habits, and food consumption during the pandemic questionnaires.

All variables which showed a *p* < 0.2 in the simple linear regression were included in the adjusted model. The stepwise method was used to simultaneously remove the weakest correlated variables and come up with a model that best explained the distribution to examine the relationship between several variables simultaneously and to eliminate confounding factors. The normality of the residuals was assessed for the two multiple linear regressions analyses, and the independence of the residuals was verified by the Durbin-Watson test. Multicollinearity was refuted using the variance inflation factor (VIF) and the significance of the models was assessed by an ANOVA test. All analyses were controlled for sex, age, BMI, and monthly income. A *p*-value of *p* < 0.05 was considered significant.

## 3. Results

### 3.1. Sample Description

The results showed that participants that completed the online survey (*n* = 598) were on average 31.76 (12.32) years old, with 64.7% females. The majority had a higher education level (55.9%), higher income (52.3%), and working or studying in home office modality (70.75%) for up to 8 h/day (70.90%). Significant difference was found between the groups, where inactive participants had a higher level of social isolation (Table 1); no difference was found between the mean of EE score in the groups (Table 2).

### 3.2. Perception on Life Habits during the Pandemic

Most of the participants had increased stress during pandemic (67.7%) and were sleeping better (67.6%). Significant differences were found between the groups, where inactive participants had increased stress and worse sleep during the pandemic (Table S1). The mean of EE score was higher for those who increased stress and reported worsening sleep during the pandemic in both groups of physical activity levels (Table 2).

### 3.3. Perception on Body Satisfaction during the Pandemic

Most of the participants were satisfied with their body (66.6%); however, 63.2% attempted to lose weight in the past 6 months. The active group reported more attempts to lose weight, with a significant difference (Table S1). The mean of EE was higher for inactive individuals who reported attempted to lose weight, as well as for active and inactive individuals who reported body dissatisfaction (Table 2).

### 3.4. Perception on Eating Habits and Food Consumption during the Pandemic

Table S1 shows the descriptive analysis of the perceptions on eating habits and food consumption during the pandemic. Most of the participants reported that they had better eating habits during the pandemic (78.8%), decreased amount of food consumption (58.9%), increased home cooking (92%), and decreased food delivery purchase (57.5%). There was a difference for the eating habits during the pandemic variable, as the active group reported better eating habits. Highest means of EE were found among inactive individuals who reported worse eating habits, increased amount of food consumption, and increased purchase of delivery (Table 2).

In the food consumption analysis, participants reported increased consumption of refined cereals (88.6%), sweets and desserts (77.9%), and fast foods (65.1%). Vegetables (68.9%) and fresh fruits (67.9%) had a reduction in consumption during the pandemic. There were differences between all food consumption variables. The active group reported increased consumption of vegetables and fruits, while the inactive group reported increased consumption of refined cereals, fast foods, and sweets and dessert during the pandemic (Table S1). In the inactive group, highest means of EE were found for those who reported increased consumption of fast food and sweets; in the active group, a higher mean of EE was found for those who reported an increase in sweets consumption (Table 2).

### 3.5. Emotional Eating

#### 3.5.1. Physically Active Group

The mean of the emotional eating scale was 33.63 (24.80). The variables which were significant (*p* < 0.05) in the simple linear regression were increased stress perception, body dissatisfaction, weight loss attempt, worse eating habits during the pandemic, increased amount of food consumed, decreased home cooking, increased food delivery purchasing, and increased sweets and dessert consumption during the pandemic.

In the final model, increased stress perception during the pandemic (β = 6.60), body dissatisfaction (β = 12.16), and increased sweets and dessert consumption during the pandemic (β = 8.33) were associated with emotional eating in the active group. The model was controlled for age, sex, income, and BMI. The regression formed a good fit for the data F(7212) = 10.48, *p* < 0.001, which accounted for 25.7% variance in increased active group emotional eating score during the pandemic (R^2^ = 0.257). Table 3 shows the crude and adjusted models.

#### 3.5.2. Physically Inactive Group

The mean of the inactive group emotional eating scale was 34.92 (26.13). In addition, increased stress, worse sleep, body dissatisfaction, weight loss attempt, worse eating habits, increased amount of food consumed, increased food delivery purchasing, and increased consumption of sweets and desserts and fast food were found in the simple linear regression to be significantly associated with emotional eating of the less active group (*p* < 0.05).

In the final model, working >8 h/day (β = 5.99) increased stress during the pandemic (β = 7.52), worse sleep (β = 6.39), body dissatisfaction (β = 5.62), increased amount of food consumption (β = 14.96), increased purchase of delivery (β = 5.89), increased vegetables (β = 7.12), and sweets and desserts (β = 6.01) consumption during the pandemic were associated with emotional eating in the inactive group. The model was controlled for age, sex, income, and BMI. The regression formed a good fit for the data F(12,345) = 18.22, *p* < 0.001, which accounted for 38.8% of variance in increased inactive group emotional eating score during the pandemic (R^2^ = 0.388). Table 4 shows the crude and adjusted models.

## 4. Discussion

Our main result was that the active individuals had fewer factors associated with EE such as body dissatisfaction, increased stress, and increased consumption of sweets and deserts during the pandemic. The inactive group had more factors associated with EE, in addition to those found among active individuals, increased amount of food consumed, increased consumption of vegetables, increased food delivery purchasing, worse sleep perception, working/studying >8 h/day.

Body dissatisfaction, perception of stress, and consumption of sweets were associated with EE regardless of the individuals’ physical activity level. Recent studies have shown a significant increase in their prevalence during the pandemic [28,51,56], and were also associated with EE [42]. Additionally, they were recognized factors associated with EE before the pandemic [41,57,58,59].

It is known that body dissatisfaction is a risk factor for eating disorders [60]. A study carried out with physically active women showed that body dissatisfaction would lead to greater physical exercise in this group, and that they would be 3.52 times more likely to develop eating disorders compared to women who were satisfied with their body [61]. A study using a convergent mixed methods carried out with athletes during the pandemic showed that the worst body image was related to eating more or eating as a result of stress. In addition to these findings, the athletes also reported that food became a stress in their lives during this period [62]. Thus, it is noticeable that there are factors during the pandemic which are strongly related to emotional eating, regardless of the physical activity or exercise level performed by the individual.

However, the fact that the individual is active can be protective for developing factors associated with EE, as reported in the present study, where variables such as worse sleep perception and working or studying >8 h/day were associated with EE only in the inactive group during the pandemic; these factors were already associated with EE before the pandemic [63,64]. Furthermore, they are also associated with stress [65,66], which is one of the main mental health problems caused by the pandemic [18]. One study showed that the association between physical inactivity and poorer mental health is partially mediated by poorer sleep quality in Brazilians during the pandemic [67], thus showing the intrinsic relationship between these three factors.

There were also factors associated with EE between inactive group, variables related to eating habits, and increasing the amount of food consumed during the pandemic, which has been associated with EE in previous studies [42,68]. There was a 10% increase in the use of delivery apps in Brazil during the pandemic [69], and it is known that these applications are characterized as obesogenic because they offer large amounts of ultra-processed foods rich in sugar and fat [70]. Furthermore, these apps intensified their marketing strategies during the pandemic to encourage consumers to purchase these foods [71], and successfully, since a study conducted in the United States showed that individuals are more motivated to wait, work, and pay more for fast food, sweets, and desserts delivery during the pandemic [72], which in turn also had their consumption increased and associated with EE during the pandemic [42,51].

Surprisingly, increased vegetable consumption was also a variable associated with EE of the inactive individuals in the present study. Similar data were observed in a study which showed that more stressed participants crave larger portions of vegetables and are more willing to order more of these foods compared to their less stressed peers [72]. In addition, a cohort study in Brazil showed an increase in vegetable consumption during the pandemic [73]. In the present study, we hypothesized that this fact occurred due to the activation of Compensatory Health Beliefs [74]. According to this theory, individuals tend to believe that unhealthy behavior (i.e., increased consumption of sweet foods and physical inactivity) can be compensated or counteracted after engaging in healthy behavior (i.e., increasing vegetable consumption); this compensatory mechanism decreases the intentions to resist unhealthy food and has already been negatively associated with the practice of physical activity [74,75]. Contrary to our results, but not with different ideas, another study showed that the consumption of unhealthy snacks led to greater engagement in physical activity [76], corroborating the hypothesis that both physical activity and eating can be part of compensatory strategies.

Finally, regarding the explanatory power of the adjusted model of the present study, it is important to emphasize that the R^2^ values of both groups showed percentages similar to those found in other articles previously carried out. Before the pandemic, studies showed R^2^ values between 29–43% in their models using EE as the dependent variable [77,78]. During the pandemic, a study that evaluated the factors associated with EE in Saudi women showed R^2^ values lower than 10% in its adjusted models [42]. However, another study carried out in the United Kingdom that aimed to assess factors associated with EE during lockdown found explanatory values of 75%; the authors discuss the strong association between the variables inserted in the model (EE before and after lockdown), and this may have increased the explanatory values of the studied regression model [79]. It is also important to emphasize that studies in the field of nutrition, even using multiple linear regression models with many predictors, present adjusted R^2^ values between 30–60% due to the high complexity of explaining what determines factors associated with food-related health outcomes [80].

### 4.1. Strengths and Limitations

This is the first study to compare factors associated with EE between active and inactive individuals during the pandemic. Previous studies have shown factors associated with EE in the general population, and it is important to explore factors associated with EE in different groups, since this dysfunctional behavior tends to occur with an increase in negative feelings such as stress. This in turn increased in its prevalence during the pandemic [21], and when added to the high prevalence of physical inactivity [81], can act synergistically to trigger or aggravate dysfunctional eating behaviors. Although our results are consistent with studies carried out before and during the pandemic, we can mention some limitations. The cross-sectional characteristic of the study does not allow us to infer causality in the results; thus, longitudinal studies and clinical trials between active and inactive individuals are necessary to more precisely identify which factors are associated with each studied group. The sampling method used lacks generalization power to the entire population, as most of the sample has a high level of education and internet access, and different contexts between individuals or countries can generate different results for active and inactive individuals.

Finally, we also cite the use of self-reported variables to analyze weight, height, and perceptions of habits and behaviors on an online platform. Although these limitations are common in behavioral science studies carried out during the pandemic [79,82], online surveys are considered a promising method to assess and track knowledge and perceptions amid outbreaks of infectious diseases [83]. We believe that these limitations were mitigated with an in-depth analysis of the questions and answers during the pre-test phase of the questionnaire.

### 4.2. Recommendations for Future Practice and Research

As well as fighting COVID-19, it is important to implement strategies that encourage individuals to have healthy lifestyle habits, implementing physical activity, stress reduction, and healthy eating into their routine. Exploring the factors associated with EE in active and inactive individuals during the pandemic helps to identify habits which are related to this behavior. This identification becomes even more relevant, as evidence shows an increase in physical inactivity, stress and worsening in the eating pattern during the pandemic [10,18], which are all recognized factors associated with EE. A robust body of evidence suggests that physical activity practice is considered effective for preventing chronic non-communicable diseases, protecting mental health and EE [22,39,40,41,84,85]. However, this practice can be used as a compensatory strategy for unhealthy eating habits [74,76], depending on the context in which the individual is inserted. All of these factors could potentially be aggravated by the pandemic. Thus, it is relevant to create strategies to combat pandemic-related disorders so that side effects related to COVID-19 do not extend longitudinally [86]. Therefore, the results of the present study are particularly useful to nutritionists, as they will help them to identify these factors and their relationship with EE and different physical activity levels in order to formulate guidance strategies to change the behavioral pattern and effectively improve the relationship with food.

## 5. Conclusions

Our results show that active individuals have fewer factors associated with EE than inactive individuals during the pandemic, regardless of age, gender, BMI, and income. This demonstrates the potential protective role of physical activity in the appearance of factors associated with EE during the COVID-19 pandemic. More studies are needed to analyze the causal and longitudinal relationship of our findings.

## Figures and Tables

**Table 1:** Descriptive analysis by level of physical activity. Brazil, 2021 (*n* = 598).

Variables		Active Group (*n* = 220)	Inactive Group (*n* = 378)	*p*
		Mean ± SD		
Age (years) ^a^		31.63 ± 11.63	31.83 ± 12.72	0.632
BMI (kg/m^2^) ^a^		24.57 ± 4.48	24.25 ± 4.84	0.223
Emotional Eating ^a^		33.63 ± 24.80	34.92 ± 26.13	0.786
		***n* (%)**		
Sex ^b^	Female	136 (61.8)	251 (66.4)	0.258
	Male	84 (38.2)	127 (33.6)	
Brazilian region ^b^	Northeast	207 (94.1)	352 (93.1)	0.773
	Southeast	11 (5.0)	20 (5.3)	
	Others	2 (0.9)	6 (1.6)	
Area ^b^	Capital	123 (55.9)	197 (52.1)	0.370
	Other cities	97 (44.1)	181 (47.9)	
Education Level ^b^	Lower education	87 (39.5)	177 (46.8)	0.084
	Higher education	133 (60.5)	201 (53.2)	
Marital Status ^b^	Single	125 (56.8)	215 (56.9)	0.989
	Married	95 (43.2)	163 (43.1)	
Monthly Household ^b^	Lower income	99 (45)	186 (49.2)	0.321
	Higher income	121 (55)	192 (50.8)	
Employment or Study modality ^b^	Home office	154 (70)	269 (71.2)	0.857
	Out of home	52 (23.6)	89 (23.5)	
	Not working or studying	14 (6.4)	20 (5.3)	
Hours of work/day ^b^	Up to 8 h/day	163 (74.1)	261 (69)	0.215
	>8 h/day	57 (25.9)	117 (31)	
Social Isolation Level ^b^	Low	124 (56.4)	123 (32.5)	0.007 *
	High	96 (43.6)	255 (67.5)	

^a^ Variables analyzed by the Mann-Whitney test. ^b^ Variables analyzed by Chi-squared test. * *p* < 0.05.

**Table 2:** Descriptive analysis of EE values for independent variables by level of physical activity, Brazil, 2021.

Variables		Active Group (*n* = 220)		Inactive Group (*n* = 378)	
		Mean	95% CI	Mean	95% CI
Sex	Female	37.95	33.74; 42.16	37.98 *	34.80; 41.15
	Male	28.51	23.11; 33.91	28.57	23.53; 33.60
Education Level	Lower education	37.03	31.73; 42.34	34.34	30.66; 38.03
	Higher education	32.98	28.62; 37.34	35.40	31.32; 39.47
Monthly Household	Lower income	33.76	28.64; 38.87	37.75	33.75; 41.86
	Higher income	35.12	30.16; 39.62	32.05	28.46; 35.63
Hours of work/day	Up to 8 h/day	34.34	30.26; 38.41	33.33	30.31; 36.35
	>8 h/day	34.99	29.02; 40.95	37.98	32.41; 43.56
Social Isolation Level	Low	33.14	28.05; 38.23	32.42	27.83; 37.01
	High	35.58	31.06; 40.10	36.01	32.62; 39.41
Stress Level	Decreased stress	27.06	21.88; 32.24	23.22	19.53; 26.91
	Increased stress	39.06 *	34.82; 43.30	39.67 *	36.30; 43.05
Sleep	Better sleep	31.37	27.57; 35.17	30.68	27.48; 33.89
	Worse sleep	43.60 *	36.94; 50.26	42.24 *	37.47; 47.02
Body Satisfaction	Satisfied	29.74	25.88; 33.59	29.02	26.03; 32.02
	Dissatisfied	46.42 *	40.60; 52.23	45.45 *	40.48; 50.41
Attempt to lose weight	Yes	36.21	32.53; 39.88	41.30 *	37.61; 44.99
	No	27.91	19.85; 35.96	27.47	23.70; 31.25
Eating Habits	Better eating habits	33.48	29.82; 37.14	30.68	27.75; 33.61
	Worse eating habits	41.35	33.12; 49.58	46.57 *	40.78; 52.36
Amount of Food Consumption	Decreased food amount	32.09	27.89; 36.29	24.82	21.86; 27.77
	Increased food amount	38.66	33.09; 44.24	47.69 *	43.52; 51.87
Food Preparation at Home	Decreased home cooking	49.14	35.31; 62.97	40.23	31.23; 49.23
	Increased home cooking	33.53	30.09; 36.97	34.30	31.43; 37.17
Purchase of Food Delivery	Decreased purchase	31.69	27.17; 36.20	29.00	25.78; 32.23
	Increased purchase	38.09	33.08; 43.10	42.96 *	38.51; 47.41
Vegetable Consumption	Decreased	33.24	29.21; 37.27	33.58	30.45; 36.71
	Increased	36.65	30.64; 42.65	38.36	32.80; 43.91
Fresh fruit Consumption	Decreased	32.88	28.71; 37.05	34.94	31.76; 38.12
	Increased	36.99	31.33; 42,65	34.61	29.23; 39.98
Refined Cereal Consumption	Decreased	32.51	23.20; 41.83	36.11	27.30; 44.91
	Increased	34.91	31.30; 38.52	34.74	31.87; 37.62
Sweets and dessert Consumption	Decreased	26.09	19.09; 33.09	24.70	19.81; 29.60
	Increased	37.66 *	33.94; 41.38	37.18 *	34.08; 40.29
Fast Food Consumption	Decreased	34,.45	28.93; 39.98	28.07	23.70; 32.45
	Increased	34.56	30.30; 38.82	37.89 *	34.52; 41.27

95% CI = 95% confidence interval. * Highest mean per group by 95% CI.

**Table 3:** Regression analysis of independent variables in relation to emotional eating of active group (*n* = 220).

Independent Variables	Crude Model		Adjusted Model			
	β (95% CI)	*p*	b	β (95% CI)	SE	*p*
Intercept				17.96 (5.41; 36.98)	9.65	0.064
Working/studying >8 h/day	0.65 (−6.86; 8.16)	0.865	-	-	-	-
Low level of social isolation	−2.69 (−9.34; 3.95)	0.426	-	-	-	-
Increased stress perception	14.23 (7.72; 20.74)	<0.001 *	0.13	6.60 (0.26; 12.93)	3.21	0.041
Worse sleep perception	11.85 (4.38; 19.31)	0.207	-	-	-	-
Body dissatisfaction	17.61 (10.69; 24.53)	<0.001 *	0.22	12.16 (5.41; 18.91)	3.42	<0.001
Weight loss attempt	10.00 (2.05; 17.95)	0.014 *	-	-	-	-
Worse eating habits	11.24 (1.45; 21.04)	0.025 *	-	-	-	-
Increased amount of food consumed	7.51 (0.75; 14.26)	0.030 *	-	-	-	-
Decreased home cooking	15.35 (1.98; 28.73)	0.025 *	-	-	-	-
Increased purchase of food delivery	8.65 (2.07; 15.23)	0.010 *	-	-	-	-
Increased consumption of vegetables	1.53 (−5.25; 8.31)	0.657	-	-	-	-
Increased consumption of fresh fruit	3.40 (−3.33; 10.14)	0.320	-	-	-	-
Increased refined cereals consumption	3.65 (−4.97; 12.29)	0.405	-	-	-	-
Increased sweets and desserts consumption	11.17 (4.02; 18.32)	0.002 *	0.15	8.33 (1.77; 14.89)	3.32	0.013
Increased fast food consumption	1.97 (−4.70; 8.65)	0.561	-	-	-	-

R^2^ = 0.257. * Variables analyzed by multiple linear regression (*p* < 0.2). The model was controlled for sex, age, monthly income, and BMI. b = standardized beta; β = unstandardized beta; 95% CI = 95% confidence interval; SE = standard error.

**Table 4:** Regression analysis of independent variables in relation to emotional eating of the inactive group (*n* = 358).

Independent Variables	Crude Model		Adjusted Model			
	β (95% CI)	*p*	b	β (95% CI)	SE	*p*
Intercept				−22.75 (−37.70; −7.81)	7.59	0.003
Working/studying >8 h/day	4.76 (−1.02; 10.55)	0.106 *	0.10	5.99 (1.26; 10.73)	2.40	0.013
Low level of social isolation	−2.95 (−8.57; 2.65)	0.301	-	-	-	-
Increased stress perception	16.32 (10.73; 21.91)	<0.001 *	0.13	7.52 (2.37; 12.68)	2.62	0.004
Worse sleep perception	9.67 (4.30; 15.05)	<0.001 *	0.11	6.39 (1.54; 11.25)	2.46	0.010
Body dissatisfaction	15.89 (10.64; 21.14)	<0.001 *	0.10	5.62 (0.65; 10.59)	2.52	0.027
Weight loss attempt	12.67 (7.55; 17.80)	<0.001 *	-	-	-	-
Worse eating habits	15.19 (9.41; 20.98)	<0.001 *	-	-	-	-
Increased amount of food consumed	22.32 (17.51; 27.13)	<0.001 *	0.28	14.96 (10.21; 19.71)	2.41	<0.001
Decreased home cooking	4.85 (−4.36; 14.08)	0.301	-	-	-	-
Increased purchase of food delivery	13.75 (8.60; 18.89)	<0.001 *	0.11	5.89 (1.15; 10.63)	2.40	0.015
Increased consumption of vegetables	5.35 (−0.59; 11,29)	0.077 *	0.12	7.12 (2.05; 12.20)	2.58	0.006
Increased consumption of fresh fruit	−0.81 (−6.65; 5.02)	0.784	-	-	-	-
Increased refined cereals consumption	−0.42 (−10.20; 9.35)	0.932	-	-	-	-
Increased sweets and desserts consumption	12.65 (6.01; 19.30)	<0.001 *	0.09	6.01 (0.15; 11.88)	2.98	0.044
Increased fast food consumption	9.43 (3.80; 15.06)	0.001 *	-	-	-	-

R^2^ = 0.388. * Variables analyzed by multiple linear regression (*p* < 0.2). The model was controlled for sex, age, monthly income, and BMI. b = standardized beta; β = unstandardized beta; 95% CI = 95% confidence interval; SE = standard error.

## Data Availability

The databases are available from the corresponding author upon reasonable request. The data are not publicly available due to restrictions, as they contain information that could compromise the privacy of research participants.

## Supplementary Materials

The following are available online at https://www.mdpi.com/article/10.3390/nu13113861/s1, Table S1: Descriptive analysis of independent variables by level of physical activity. Brazil, 2021 (*n* = 598).
