# Supplementary material for: Is Physical Activity Protective against Emotional Eating Associated Factors during the COVID-19 Pandemic? A Cross-Sectional Study among Physically Active and Inactive Adults
Source: Nutrients. 2021 Oct 28;13(11):3861. doi: 10.3390/nu13113861 (PMC8618670; doi:10.3390/nu13113861)
Supplement: Supplementary file 1 [file nutrients-13-03861-s001.zip › nutrients-1399812-supplementary.pdf]

Table S1 Descriptive analysis of independent variables by level of physical activity. Brazil, 2021 (n=598)

Variables	Total sample	Active group (n=220)	Inactive group (n=378)	p
		n (%)		
**Perception on life habits during the pandemic**				
**Stress Level During the Pandemic**				0.011*
Decreased stress	193 (32.3)	85 (38.6)	108 (28.6)	
Increased stress	405 (67.7)	135 (61.4)	270 (71.4)	
**Sleep During the Pandemic**				0.003*
Better sleep	404 (67.6)	165 (75)	239 (63.2)	
Worse sleep	194 (32.4)	55 (25)	139 (36.8)	
**Perception on body satisfaction**				
**Body Satisfaction**				0.057
Satisfied	398 (66.6)	157 (71.4)	241 (63.8)	
Dissatisfied	200 (33.4)	63 (28.6)	137 (36.2)	
**Attempt to lose weight**				<0.001*
Yes	378 (63.2)	173 (78.6)	205 (54.2)	
No	220 (36.8)	47 (21.4)	173 (45.8)	
**Perception on eating habits during the pandemic**				
**Eating Habits**				<0.001*
Better eating habits	471 (78.8)	192 (87.3)	279 (73.8)	
Worse eating habits	127 (21.2)	28 (12.7)	99 (26.2)	
**Amount of Food Consumption**				0.143
Decreased food amount	352 (58.9)	138 (62.7)	214 (56.6)	
Increased food amount	246 (41.1)	82 (37.3)	164 (43.4)	
**Food Preparation at Home**				0.253
Decreased home cooking	48 (8.0)	14 (6.4)	34 (9)	
Increased home cooking	550 (92.0)	206 (93.6)	344 (91)	
**Purchase of Food Delivery**				0.924
Decreased purchase of food delivery	344 (57.5)	126 (57.3)	218 (57.7)	
Increased purchase of food delivery	254 (42.5)	94 (42.5)	160 (42.3)	
**Perception on food consumption during the pandemic**				
**Vegetable Consumption**				0.002*
Decreased	412 (68.9)	135 (61.4)	277 (73.3)	
Increased	186 (31.1)	85 (38.6)	101 (26.7)	
**Fresh fruit Consumption**				0.005*
Decreased	404 (67.6)	133 (60.5)	271 (71.7)	
Increased	194 (32.4)	87 (39.5)	107 (28.3)	
**Refined Cereal Consumption**				<0.001*
Decreased	68 (11.4)	39 (17.7)	29 (7.7)	
Increased	530 (88.6)	181 (82.3)	349 (92.3)	
**Sweets and dessert Consumption**				0.003*
Decreased	132 (22.1)	63 (28.6)	69 (18.3)	

Increased	466 (77.9)	157 (71.4)	309 (81.7)	
**Fast Food Consumption**				0.004*
Decreased	209 (34.9)	93 (42.3)	116 (30.7)	
Increased	389 (65.1)	127 (57.7)	262 (69.3)	

[a] Variables analyzed by the Mann-Whitney test. *Significant variables by the Chi-squared test.
